# Serious role of non-quarantined COVID-19 patients for random walk simulations

**DOI:** 10.1038/s41598-021-04629-2

**Published:** 2022-01-14

**Authors:** Nariyuki Nakagiri, Kazunori Sato, Yukio Sakisaka, Kei-ichi Tainaka

**Affiliations:** 1 School of Human Science and Environment, University of Hyogo, Himeji, 670-0092 Japan; 2 Department of Mathematical and Systems Engineering, Shizuoka University, Hamamatsu, 432-8561 Japan; 3 Institute of Preventive and Medicinal Dietetics, Nakamura Gakuen University, Fukuoka, 814-0198 Japan; 4 Division of Early Childhood Care and Education, Nakamura Gakuen University Junior College, Fukuoka, 814-0198 Japan

**Keywords:** Computational models, Infectious diseases, Mathematics and computing

## Abstract

The infectious disease (COVID-19) causes serious damages and outbreaks. A large number of infected people have been reported in the world. However, such a number only represents those who have been tested; e.g. PCR test. We focus on the infected individuals who are not checked by inspections. The susceptible-infected-recovered (SIR) model is modified: infected people are divided into quarantined (Q) and non-quarantined (N) agents. Since N-agents behave like uninfected people, they can move around in a stochastic simulation. Both theory of well-mixed population and simulation of random-walk reveal that the total population size of Q-agents decrease in spite of increasing the number of tests. Such a paradox appears, when the ratio of Q exceeds a critical value. Random-walk simulations indicate that the infection hardly spreads, if the movement of all people is prohibited ("lockdown"). In this case the infected people are clustered and locally distributed within narrow spots. The similar result can be obtained, even when only non-infected people move around. However, when both N-agents and uninfected people move around, the infection spreads everywhere. Hence, it may be important to promote the inspections even for asymptomatic people, because most of N-agents are mild or asymptomatic.

## Introduction

The infectious disease (COVID-19) caused by the coronavirus (SARS-CoV-2) is spreading to a large number of individuals^1–3^. According to the WHO report, many people died by COVID-19^4^. It is an urgent worldwide issue to break the chain of COVID-19 infection. Two major protection approaches are known: prevention and isolation. The former is a prepared protection for a person, such as masks and hand-washing. Vaccination has been thought to be the most effective method of prevention^5,6^. In contrast, the isolation (or segregation) means the cut-off of contact between infected and non-infected people. An example is quarantine to put an infected person into hospital. Another example is lockdown: mobilities of people are prohibited^7,8^. Very recently, vaccination is found to be the most effective method for COVID-19^6,9^. However, in England, Republic of Chile and State of Arizona, the infection is widespread, even though the vaccination rate is above 40%^4,6^. Moreover, much time is still needed for vaccines to become widespread. Various types of infection control measures will be necessary.

We apply agent-based model (ABM) used in complex systems^10–17^. For infectious diseases, ABM is one of fundamental tools^18–20^. The epidemic spreading on lattices and networks have been studied by many authors^20–25^. In the present article, we explore the effect of mobility (random walk) on the isolation of infectious people. Infected people are divided into two groups, quarantined (Q) and non-quarantined (N) agents. The former can be detected by inspections; e.g. PCR test.

A certain proportion of infected people become severe, such as pneumonia^3,26^. In many countries, hospital beds are very crowded. To suppress the epidemic spreading, it should be important to quarantine the infected individuals. From the early stage of the epidemic, the importance of asymptomatic infection has been pointed out because the asymptomatic patient also behaves as a cryptic source of spreading infection^27^. Thus, a large number of people have being inspected by PCR test in the world. However, the application of PCR test for no symptom person has been restricted in Japan because the medical diagnostic PCR test resources were too poor to promote the test in the early period of the epidemic. Major academic societies in Japan claimed that PCR testing was basically not recommended for asymptomatic or mildly ill individuals^28^. Such claims raise some problems. (1) Is it okay to leave the infected person unchecked? (2) Does the number of Q-agents (PCR-positive) always increase with the number of tests? It has been reported for COVID-19 that most infected people have mild or no symptoms^3,29^. Because such people behave like uninfected people, they have considerably high infectivity^30–33^. This may be a distinct feature of COVID-19 never observed for the previous coronaviruses, SARS and MERS. We carry out simulations of random walk to report N-agents play a major role for epidemic spreading.

**Figure 1:**
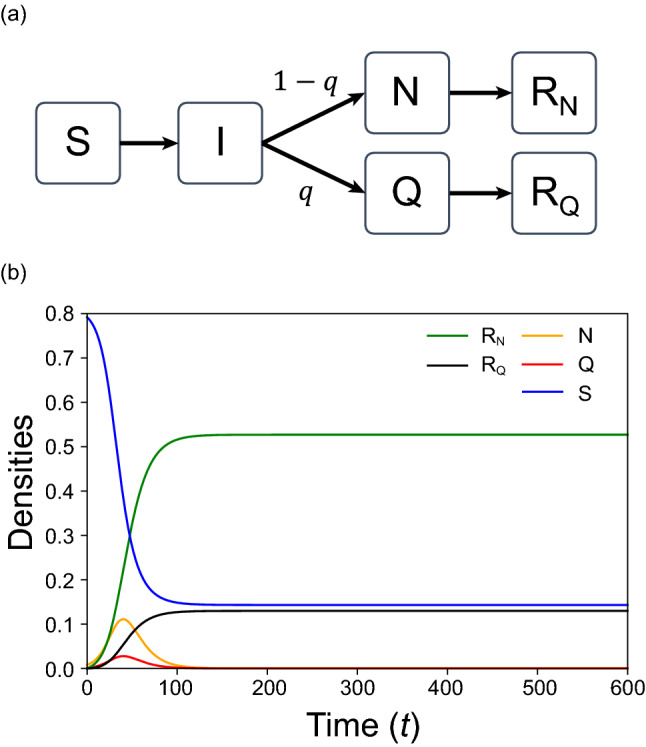
Model and population dynamics. (**a**) Schematic illustrations of infection model. (**b**) Predictions of mean-field theory (MFT) which agree with the simulation results of global interaction. In the present article, we put ${\rho }_{0}=0.2$ and the initial condition is set as follows: (*S*, *N*) = (0.792, 0.008) and the other densities are zero at *t* = 0. Values of parameters in all figures are listed in Table S1.

So far, various epidemic models have been presented for analyzing an epidemic spread^34–37^. In most cases, the epidemic of influenza and other infectious diseases has been theoretically explained by SIR model^38–41^ that considers susceptible (S), infected (I), and recovered (R) people. Interactions are represented as follows:

1a $${\text{S}} + {\text{I}} \to {\text{I}} + {\text{I}}\;\;\;\;\;\;\left( {{\text{rate}}:\beta } \right)$$

1b $${\text{I}} \to {\text{R}}\;\;\;\;\;\;\left( {{\text{rate}}:\gamma } \right)$$

where *β* and *γ* represent infection and recovery rates, respectively. The spatial and network versions of SIR model is studied extensively in various fields^42–46^. In the present paper, we modify the SIR model as shown in Fig. 1a. Infected people are divided into quarantined (Q) and non-quarantined (N) agents. Similarly, the recovered (plus dead) people are also divided into ${\mathrm{R}}_{\mathrm{Q}}$ and ${\mathrm{R}}_{\mathrm{N}}$. Such a division may give us valuable information, because only the population size of Q (or ${\mathrm{R}}_{\mathrm{Q}}$) is announced in public. It is important to know the behavior of N.

## Method

We study epidemic spreading on a lattice. Each cell is either empty (O) or occupied by an individual (agent). The agent takes one of five states: S, Q, N, ${\mathrm{R}}_{\mathrm{Q}}$ and ${\mathrm{R}}_{\mathrm{N}}$. Symbols and parameters are listed in Table 1. Interactions are represented as follows:

2a $${\text{S}} + {\text{N}} \to i + {\text{N}}\;\;\;\;\;\;\;({\text{rate}}:\beta_{{\text{N}}} )$$

2b $${\text{S}} + {\text{Q}} \to i + {\text{Q}}\;\;\;\;\;\;({\text{rate}}:\beta_{{\text{Q}}} )$$

2c $${\text{N}} \to {\text{R}}_{{\text{N}}} \;\;\;\;\;({\text{rate}}:\gamma_{{\text{N}}} )$$

2d $${\text{Q}} \to {\text{R}}_{{\text{Q}}} \;\;\;\;\;({\text{rate}}:\gamma_{{\text{Q}}} )$$

where $i$ denotes an infected agent ($i=\mathrm{N},\mathrm{ Q}$). The parameters ${\beta }_{\mathrm{i}}$ and ${\gamma }_{\mathrm{i}}$ denotes the infection and recovery rates of $i$, respectively. We introduce "quarantine ratio" $q$ which denotes the ratio of Q among all infected agents (see Fig. 1a). If $q=1$, every infected agent is confirmed to be infected by testing (e.g. PCR test). In contrast, if $q=0$, no infected agents are tested. We consider both ${\mathrm{R}}_{\mathrm{N}}$ and ${\mathrm{R}}_{\mathrm{Q}}$ have no infectivity. When the agent Q stays in hospital, ${\beta }_{\mathrm{Q}}$ takes a negligible value. However, when Q is waiting (or staying) at home, it is not negligible. In the present article, we assume ${\beta }_{\mathrm{N}}>{\beta }_{\mathrm{Q}}$ as discussed later.

**Table 1:** Descriptions of symbols and parameters.

Symbols and parameters	Descriptions
S	Susceptible agent
Q	Quarantined agent
N	Non-quarantined agent
Agent $i$	Infected agent ($i=\mathrm{Q},\mathrm{ N}$)
${\mathrm{R}}_{\mathrm{Q}}$	Recovered (or died) agent from Q
${\mathrm{R}}_{\mathrm{N}}$	Recovered (or died) agent from N
${\beta }_{N}$	Infection rate of N
${\beta }_{Q}$	Infection rate of Q
${\gamma }_{N}$	Recovery rate of N
${\gamma }_{Q}$	Recovery rate of Q
${\rho }_{0}$	Initial density of empty cells
$q$	Quarantine ratio of Q to the sum of N and Q agents
Agent $j$	($j=\mathrm{S},\mathrm{N},\mathrm{ Q}, {\mathrm{R}}_{\mathrm{N}}, {\mathrm{R}}_{\mathrm{Q}}$)
${m}_{j}$	Migration rate of agent $j$
$t$	Time [MCS]
${R}_{Q}(\infty )$	Final density of ${\mathrm{R}}_{\mathrm{Q}}$ for $t\to \infty$
${R}_{N}(\infty )$	Final density of ${\mathrm{R}}_{\mathrm{N}}$ for $t\to \infty$
${q}_{MAX}$	Value of $q$ at the maximum of ${R}_{Q}(\infty )$

Simulations are carried out either by local or global interaction. Initially, few infected individuals are randomly positioned, and we put empty cells with density ${\rho }_{0}$; in this article we put ${\rho }_{0}=0.2$. Simulation for local interaction is performed as follows.

(i) Infection processes (2a) and (2b): we randomly choose a single cell. If the cell is S and its nearest-neighbor site is occupied by N or Q, then S change to infected agent $i$ with probability ${\beta }_{i}$. We put $i$=Q by probability $q$, but $i$=N by probability $(1-q)$. Here, boundaries are periodic.
(ii) Recovery and death processes (2c) and (2d): we randomly select one cell. If the site is N, it changes to ${\mathrm{R}}_{\mathrm{N}}$ with rate ${\gamma }_{\mathrm{N}}$. Similarly, if the selected site is Q, it becomes ${\mathrm{R}}_{\mathrm{Q}}$ with rate ${\gamma }_{\mathrm{Q}}$.
(iii) Random walk: we randomly select two neighboring cells. If the first and second chosen cells are respectively the cell of agent $j$ and empty cell ($j=\mathrm{S},\mathrm{N},\mathrm{Q},{\mathrm{R}}_{\mathrm{N}},{\mathrm{R}}_{\mathrm{Q}}$), then both cells are exchanged with migration rate ${m}_{j}$. Hence, the agent $j$ moves into the empty cell with rate ${m}_{j}$. Note that we have ${m}_{j}=2$, when only step (iii) is repeated twice.

In the simulation, the unit of time $t$ is measured by Monte Carlo step (MCS)^14,15^. Namely $t$ increases by 1 MCS, when steps (i)–(iii) are repeated by 10^4^ times; note 10^4^ is the total cell number of lattice. The simulation is continued until the system reaches a steady state. In the case of global interaction, infection process (i) occurs between any pair of cells. We can skip the random walk in the simulation of global interaction, because all agents randomly distribute.

The well-mixed population for epidemic model is given by mean-field theory (MFT):

3a $$dS/dt=-{\beta }_{N}SN-{\beta }_{\mathrm{Q}}SQ$$

3b $$dN/dt=(1-q){\beta }_{\mathrm{N}}SN+(1-q){\beta }_{\mathrm{Q}}SQ-{\gamma }_{N}N$$

3c $$\mathrm{dQ}/\mathrm{dt}=q{\beta }_{\mathrm{N}}SN+q{\beta }_{\mathrm{Q}}SQ-{\gamma }_{\mathrm{Q}}Q$$

3d $$d{R}_{N}/dt={\gamma }_{\mathrm{N}}N$$

3e $$d{R}_{Q}/dt={\gamma }_{\mathrm{Q}}Q$$

Here the densities of S, N, Q, ${\mathrm{R}}_{\mathrm{N}}$ and ${\mathrm{R}}_{\mathrm{Q}}$ are shown in their italics. The total densities of agents are given by $(1-{\rho }_{0})$, where ${\rho }_{0}$ is the density of empty cell. If $q=0$ and ${\beta }_{\mathrm{Q}}=0$, then Eqs. (3a–3e) agrees with those for SIR model. The threshold phenomenon is well known for SIR model^34,38^. When ${\beta }_{N}/{\gamma }_{N}$ > 1/S(0), the disease spreads. Similarly, if $q=1$ and ${\beta }_{\mathrm{N}}=0$, the disease spreads for ${\beta }_{\mathrm{Q}}/{\gamma }_{\mathrm{Q}}$ > 1/S(0).

## Results

### Results for global interaction

The simulation results of global interaction agree with those predicted by MFT. This is because the global interaction corresponds to the assumption of well-mixed population. First, the numerical calculation for global interaction is reported. In Fig. 1b, a typical population dynamics for MFT are displayed. Model parameters used in all figures are listed in Table S1 (see Supplementary file). At the final equilibrium ($t\to \infty$), both densities *N* $(\infty )$ and *Q* $(\infty )$ become zero, but ${R}_{N}(\infty )$ and ${R}_{Q}(\infty )$ take constant values. Namely, agents N and Q always change to ${\mathrm{R}}_{\mathrm{N}}$ and ${\mathrm{R}}_{\mathrm{Q}}$, respectively. Hereafter, we will call ${R}_{N}\left(\infty \right)+{R}_{Q}\left(\infty \right)$ "total infection" and ${R}_{Q}(\infty )$ "apparent infection". The former accurately indicates the degree of infection, but its measurement may be impossible. Realistically, only the latter index is announced in public.

**Figure 2:**
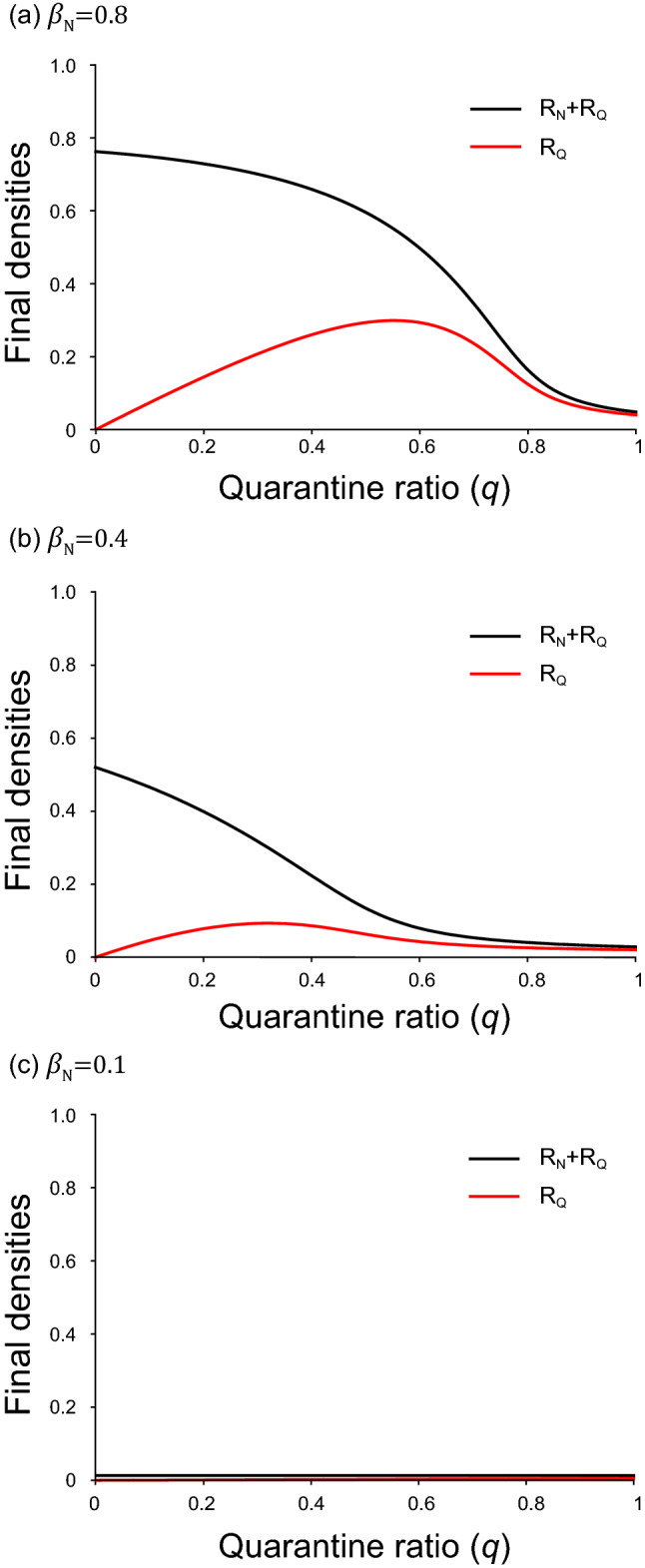
Results of final densities for MFT. The total infection [${R}_{N}\left(\infty \right)+{R}_{Q}\left(\infty \right)$] and apparent infection [${R}_{Q}\left(\infty \right)$] are plotted against quarantine ratio (*q*). We fix ${\gamma }_{\mathrm{N}}={\gamma }_{\mathrm{Q}}=0.2$, ${\beta }_{\mathrm{Q}}=0.1$. (**a**) ${\beta }_{\mathrm{N}}=0.8$, (**b**) ${\beta }_{\mathrm{N}}=0.4$ and (**c**) ${\beta }_{\mathrm{N}}=0.1$.

In Fig. 2, both total and apparent infections are depicted against the ratio $q$. We find a threshold phenomenon as observed for SIR model^34,38^. When the ratio ${\beta }_{N}/{\gamma }_{N}$ takes a small value, the disease never spreads. In contrast, when ${\beta }_{\mathrm{N}}/{\gamma }_{\mathrm{N}}$ takes a large value, the infection can spread. The threshold of ${\beta }_{N}/{\gamma }_{N}$ becomes small, when ${\beta }_{\mathrm{Q}}/{\gamma }_{\mathrm{Q}}$ takes a large value. We also find that the apparent infection (${R}_{Q}$) takes a maximum value at $q={q}_{MAX}$, where ${0<q}_{MAX}<1$. When ${q<q}_{MAX}$, the total number of Q increases with increasing *q*. On the contrary, when ${q>q}_{MAX}$, it decreases in spite of the increase of *q*. The value of ${q}_{MAX}$ is found to be increased with the increase of ${\beta }_{N}/{\gamma }_{N}$. It should be emphasized that the total infection monotonically decreases with increasing *q*. Hence, isolating the infected agents is effective to suppress the infection. In this paper, we put ${\gamma }_{N}{=\gamma }_{\mathrm{Q}}$; this is because both ratios ${\beta }_{N}/{\gamma }_{N}$ and ${\beta }_{\mathrm{Q}}/{\gamma }_{\mathrm{Q}}$ are found to be more important parameters than ${\gamma }_{N}$ and ${\gamma }_{\mathrm{Q}}$. Numerical calculation reveals that both total and apparent infections increase with the increase of either ${\beta }_{N}/{\gamma }_{N}$ or ${\beta }_{\mathrm{Q}}/{\gamma }_{\mathrm{Q}}$.

### Results for random-walk simulation

**Figure 3:**
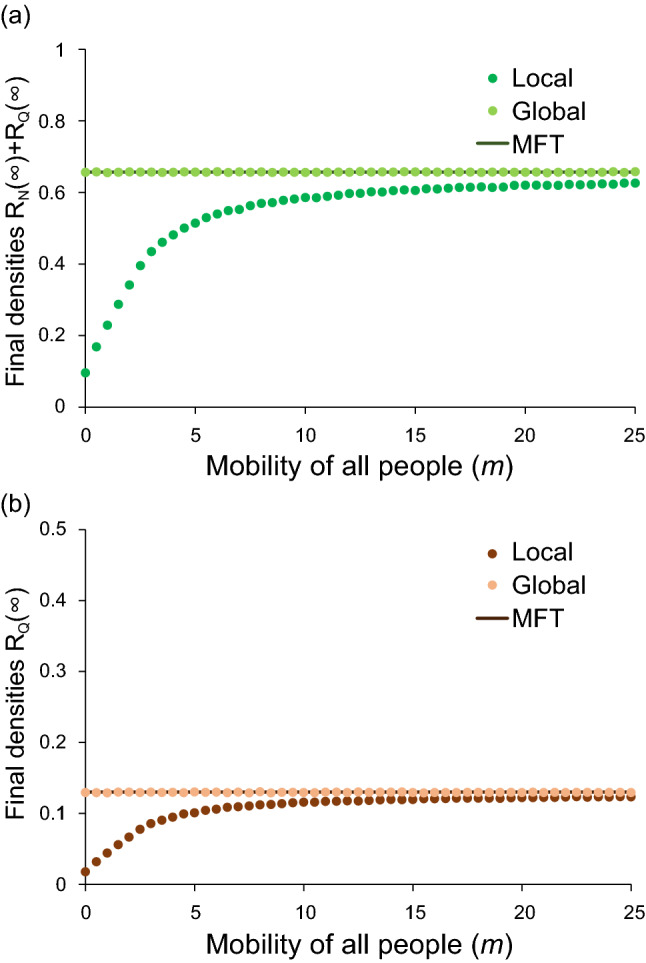
Results of random-walk simulation at the final stage ($t=1000$). All people (agents) randomly migrate with the same migration rate (*m*) on a lattice ($100\times 100$). In (**a**) and (**b**), the total infection [${R}_{N}\left(\infty \right)+{R}_{Q}\left(\infty \right)$] and apparent infection [${R}_{Q}\left(\infty \right)$] are plotted against *m*, respectively. The straight lines indicate the results of MFT which agree with those of global interaction.

Simulation results for local interaction are described. To know the relation between local and global simulations, we first assume the special case that the migration rate ($${m}_{j})$$ of agent $$j$$ takes the same value for all agents ($${m}_{j}=m$$ for $$j=\mathrm{S},\mathrm{N},\mathrm{Q},{\mathrm{R}}_{\mathrm{N}},{\mathrm{R}}_{\mathrm{Q}}$$). In Fig. 3, the effect of random walk is illustrated; in (a) and (b), the final densities are plotted against the migration rate ($$m$$). It is found that both total and apparent infections increase with $$m$$. The infection hardly spreads for $$m=0$$, while it widely spreads for a large value of $$m$$. Especially when $$m$$ is sufficiently large, the results of local interaction approach those predicted by MFT.

**Figure 4:**
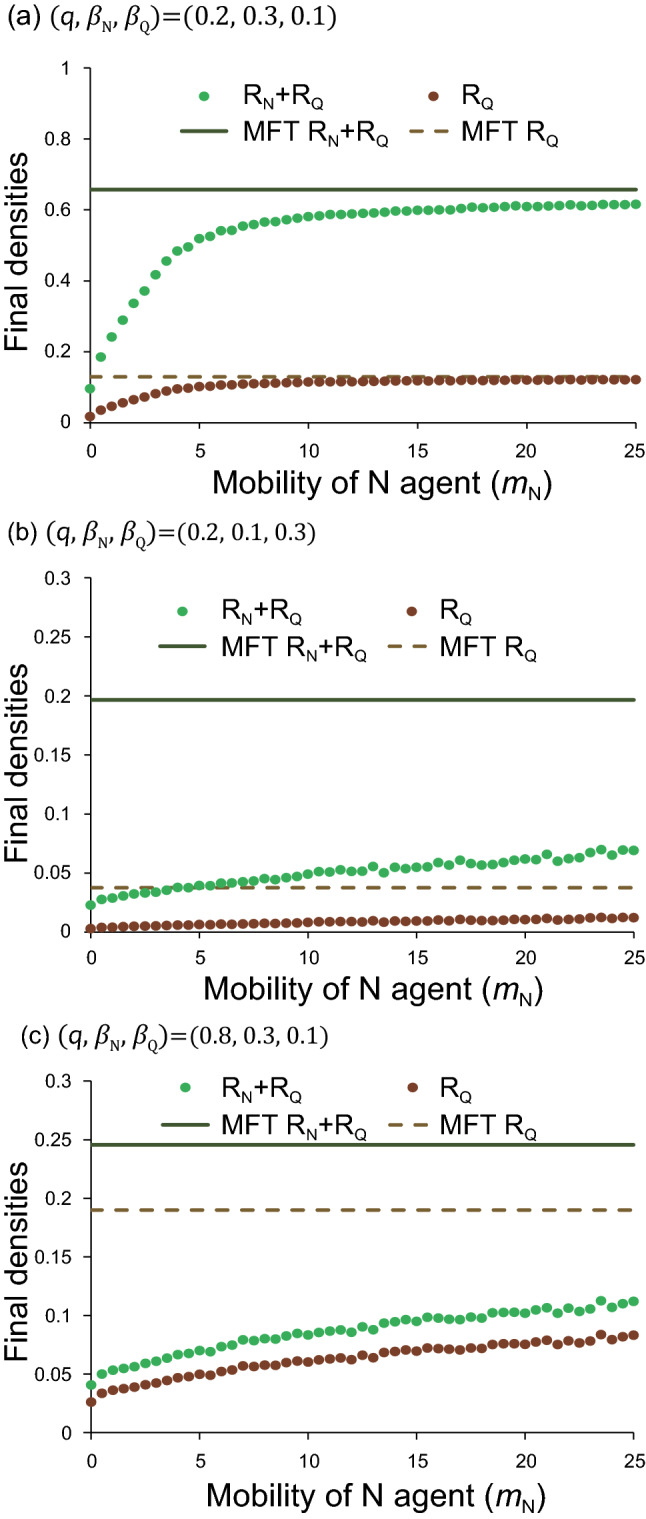
Serious effect of non-quarantined agents (N). Both [${R}_{N}\left(\infty \right)+{R}_{Q}\left(\infty \right)$] and ${R}_{Q}\left(\infty \right)$ are plotted against ${m}_{\mathrm{N}}$. Uninfected agent (S) can move with ${m}_{\mathrm{S}}=2$. The horizontal axis means the migration rate (${m}_{\mathrm{N}})$ of N, where both N and ${\mathrm{R}}_{\mathrm{N}}$ move with the same rate. In contrast, the other agents (Q and ${\mathrm{R}}_{\mathrm{Q}}$) never move: ${m}_{k}=0$ for $k=\mathrm{Q},{\mathrm{R}}_{\mathrm{Q}}$.

Realistically, both agents Q and ${\mathrm{R}}_{\mathrm{Q}}$ never move. Next, we consider the case that three agents (S, N, ${\mathrm{R}}_{\mathrm{N}}$) can move; we fix ${m}_{\mathrm{S}}=2$, and change the migration rates of N and ${\mathrm{R}}_{\mathrm{N}}$ with the same rate (${m}_{k}={m}_{\mathrm{N}}$ for $k=$ ${\mathrm{R}}_{\mathrm{N}}$). In Fig. 4, the final densities are plotted against ${m}_{\mathrm{N}}$, where (a) $\left(q,{\beta }_{N},{\beta }_{Q}\right)=\left(0.2, 0.3, 0.1\right)$, (b) $(q,{\beta }_{N},{\beta }_{Q})=(0.2, 0.1, 0.3)$ and (c) $(q,{\beta }_{N},{\beta }_{Q})=(0.8, 0.3, 0.1)$. Both values $q=0.2$ and $q=0.8$ represent the cases that the inspection is insufficient and sufficient, respectively. Figure 4b represents a symmetrical case to Fig. 4a: ${\beta }_{N}<{\beta }_{Q}$. It is found from Fig. 4a that both total infection (${R}_{N}+{R}_{Q}$) and apparent infection (${R}_{Q}$) rapidly increase with the increase of ${m}_{\mathrm{N}}$. When N and ${\mathrm{R}}_{\mathrm{N}}$ sufficiently move around, the simulation results of random walk agree with those predicted by MFT (well-mixed population). The infection rapidly spreads. In contrast, Fig. 4b shows different behavior. For small values of ${\beta }_{\mathrm{N}}$, both total and apparent infections hardly increase in spite of the increase of ${m}_{\mathrm{N}}$; the infection becomes very difficult to spread. Similarly, when the inspection is sufficient ($q=0.8$), the infection can be suppressed.

**Figure 5:**
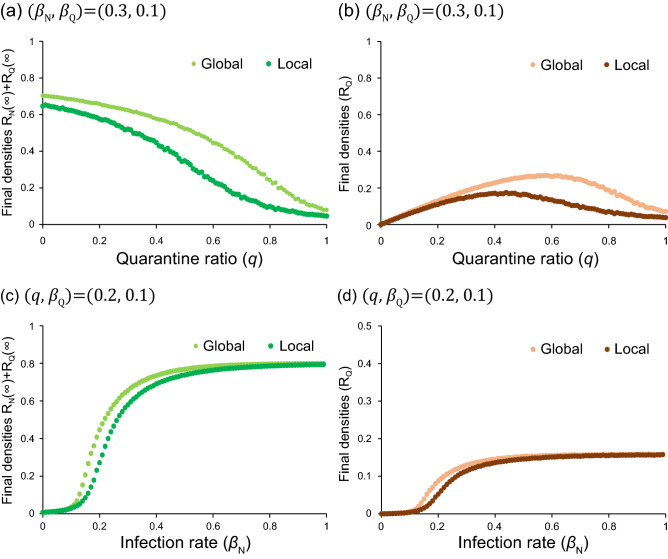
Comparison between local and global interactions. In the former, random-walk simulations are carried out; three agents (S, $\mathrm{N}, {\mathrm{R}}_{\mathrm{N}}$) can move with the same rate ${m}_{j}=10$ for $j=\mathrm{S}, \mathrm{N}, {\mathrm{R}}_{\mathrm{N}}$ (${m}_{k}=0$ for $k=\mathrm{Q},{\mathrm{R}}_{\mathrm{Q}}$). In (**a**) and (**b**), both [${R}_{N}\left(\infty \right)+{R}_{Q}\left(\infty \right)$] and ${R}_{Q}\left(\infty \right)$ are plotted against the quarantine ratio (*q*), respectively. In (**c**) and (**d**), the same as (**a**) and (**b**) are plotted, but the horizontal axis denotes the infection rate (${\beta }_{\mathrm{N}}$) of N.

**Figure 6:**
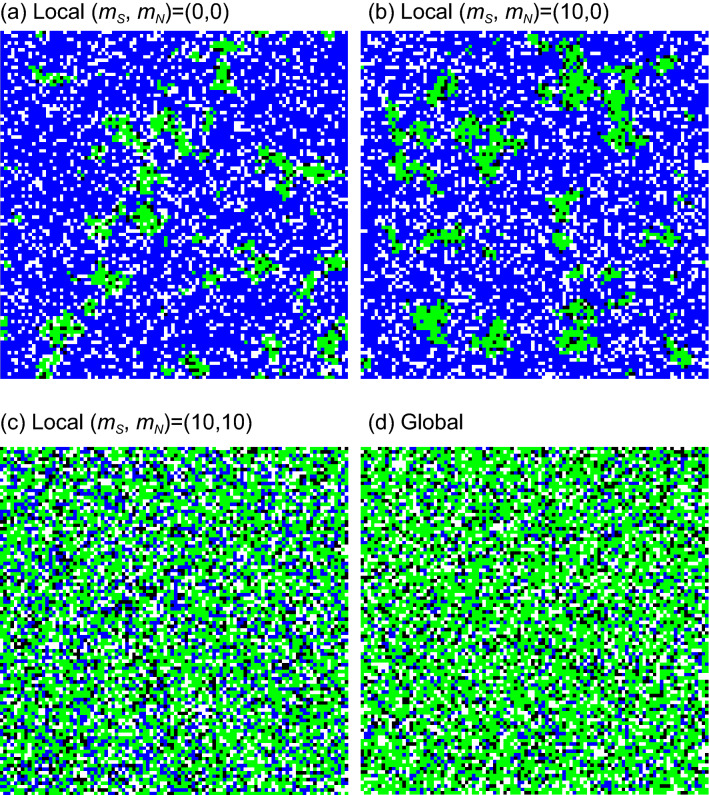
Typical spatial patterns at the final stage ($t=1500$). (**a**) Interactions occur between adjacent cells (local interaction). Nobody can move around ("lockdown"). (**b**) Local interaction. Only agent S can move around (${m}_{\mathrm{S}}=10$, but ${m}_{j}=0$ for $j\ne \mathrm{S}$). (**c**)Local interaction. Mobile agents are limited to S, N and ${\mathrm{R}}_{\mathrm{N}}$; namely ${m}_{j}=10$ for $j=\mathrm{S},\mathrm{N},{\mathrm{R}}_{\mathrm{N}}$ but ${m}_{k}=0$ for $k=\mathrm{Q},{\mathrm{R}}_{\mathrm{Q}}$). (**d**) Global interaction (random distribution). Each site is either empty (white) or occupied by S (blue), R_N_ (green) or R_Q_ (black).

In Fig. 5a and b, the total (${R}_{N}+{R}_{Q}$) and apparent (${R}_{Q}$) infections are plotted against *q*, respectively. Here, three agents ($\mathrm{S},\mathrm{N},{\mathrm{R}}_{\mathrm{N}}$) can move: ${m}_{j}=10$ for $j=\mathrm{S},\mathrm{N},{\mathrm{R}}_{\mathrm{N}}$ but ${m}_{k}=0$ for $k=\mathrm{Q},{\mathrm{R}}_{\mathrm{Q}}$. As predicted by MFT, the total infection monotonically decreases with the increase of *q*, but the apparent infection has the maximum at $q={q}_{MAX}$. The value of ${q}_{MAX}$ for local interaction is found to be smaller, compared to the prediction of MFT. In Fig. 5c and d, both total and apparent infections are also plotted against ${\beta }_{\mathrm{N}}$, respectively. These figures display the phase transition. When ${\beta }_{\mathrm{N}}$ takes a small value, the infection never spreads. With the increase of ${\beta }_{\mathrm{N}}$, the infected people suddenly increase. In Fig. 6, typical spatial distributions are displayed, where no agent moves in (a), only S moves in (b), and three agents (S, $\mathrm{N}, {\mathrm{R}}_{\mathrm{N}}$) move in (c). For the sake of comparison, the result of global simulation (random distribution) is displayed in Fig. 6d. In the cases of Fig. 6a and b, the infection is suppressed; many cells are occupied by blue (S). The infected agents form clusters and stay inside localized spots. However, in Fig. 6c and d, the infection widely spread. It is therefore important to stop the movement of N agents.

## Discussion

The coronavirus SARS-CoV-2 has distinct features never seen for previous coronaviruses, such as SARS and MERS. In the case of SARS-CoV-2, many infected people have mild or asymptomatic symptoms, but they may have considerably high infectivity^27,30–33^. We demonstrate the serious role of infected people who are not quarantined (N). To this end, we have modified SIR model; infected individual (I) is divided into two groups (N and Q). Similarly, recovered individual (R) is divided into ${\mathrm{R}}_{\mathrm{N}}$ and ${\mathrm{R}}_{\mathrm{Q}}$ to distinguish the total and apparent infections. Model (2) resembles SIQR model^24,43,44^. In the latter case, infected agent (I) always transitions to Q with a constant probability (per unit time). For this reason, N cannot be defined well. However, in model (2), we assume the transition occurs only in the early stages of infection. Those who did not transition to Q are defined by N agents; in simple terms, a person who is infected but not tested is an agent N.

The setting of parameter values is discussed. If the value of ${\beta }_{N}/{\gamma }_{N}$ or ${\beta }_{Q}/{\gamma }_{Q}$ is sufficiently high, then the infection easily spreads. In the present paper, the parameters are set based on the following facts: (i) COVID-19 has been suppressed due to lockdown and other regulation of people’s behavior. (ii) Once unlocked, the infection spreads again (e.g. USA and Australia)^4^. In our model, the disease disappears when all people stop moving, but the infection spreads when people move frequently (see Fig. 3). Moreover, we assume ${\beta }_{\mathrm{N}}>{\beta }_{\mathrm{Q}}$ for the following reasons. Asymptomatic or mildly infected individuals tend to become agent N. On the other hand, those with severe disease may become agent Q. Since agent Q is quarantined, its infectivity (${\beta }_{\mathrm{Q}}$) may be low. In contrast, the infectivity (${\beta }_{\mathrm{N}}$) of N may be higher than ${\beta }_{\mathrm{Q}}$. This is because the agent N looks like uninfected agent (S); nevertheless, the value of ${\beta }_{N}/{\gamma }_{N}$ may considerably high^27,30–33^. Provided that ${\beta }_{\mathrm{N}}$ takes a small value, the infection hardly spreads as shown in Fig. 4b.

Random-walk simulation reveals that both total [${R}_{N}\left(\infty \right)+{R}_{Q}\left(\infty \right)$] and apparent [${R}_{Q}(\infty )$] infections rapidly increase with increasing the mobility (${m}_{N}$) of N (see Fig. 4). However, when the value of ${\beta }_{N}/{\gamma }_{N}$ is low, or when $q$ takes a high value, the infection hardly spreads. Such a low value of ${\beta }_{N}/{\gamma }_{N}$ means the weak infectivity of N, and the high value of $q$ denotes the small population size of N. Hence, non-quarantined infected individuals (N) play serious roles for epidemic spreading. It should be noted that the movement of ${\mathrm{R}}_{\mathrm{N}}$ never makes a large difference: if ${\mathrm{R}}_{\mathrm{N}}$ stops to move, Figs. 4, 5, and 6 are almost unchanged. Only when the movement of N is suppressed, the infection can be suppressed.

We discuss spatial pattern formations (see Fig. 6). As illustrated in Fig. 6a, the infection hardly spreads, because all people never move ("lockdown"). Most cells are blue (agent S). The infected agents (green and black) stay inside localized spots. Such a cluster formation may be a merit of lockdown: it is advantageous in taking measures against infectious diseases. Similarly, even when only agent S (non-infected person: blue cell) moves, the infection can be suppressed (see Fig. 6b). The following question arises: Why the infection is suppressed, despite the large number of non-infected persons intensely move. We consider this suppression comes from the spatial pattern formation. Both Fig. 6a and b have the similar distributions: the infected people form clusters. Since infected agents aggregate, the contact between infected and non-infected individuals is effectively decreased.

## Conclusion

In the present article, we demonstrate the serious role of infected people who are not quarantined (N). Both mean-field theory and Monte Carlo simulation reveal the result schematically shown in Fig. S1 (see Supplementary file). The total infection monotonically decreases with the increase of quarantine ratio (*q*). In contrast, the apparent infection has the maximum at ${q=q}_{\mathrm{MAX}}$. For ${q>q}_{\mathrm{MAX}}$, the density of infected people decreases in spite of increasing *q.* Hence, it is important to promote the inspections; e.g. PCR test. Random-walk simulation reveals that the infection rapidly spreads with increasing the mobility of N. If the movement of N is suppressed, the infection can be suppressed. This conclusion is also confirmed by spatial distribution. Figure 6a indicates the spatial pattern of "lockdown"; infected people form clusters, because all people cannot move. Similar distribution is observed, if infected agents never move (see Fig. 6b). Since infected people aggregate, the infection hardly spreads. Hence, suppressing the movement of infected people (or expanding the tests) is as effective as lockdown. This result for COVID-19 should be unique property never seen for both SARS and MERS. It is therefore important to detect and quarantine the asymptomatic SARS-CoV-2 infected persons^27,30–33^.

## Supplementary Information

Supplementary Information.
