# Supplementary material for: Serious role of non-quarantined COVID-19 patients for random walk simulations
Source: Sci Rep. 2022 Jan 14;12:738. doi: 10.1038/s41598-021-04629-2 (PMC8760292; doi:10.1038/s41598-021-04629-2)
Supplement: Supplementary file 1 — Supplementary Information. [file 41598_2021_4629_MOESM1_ESM.pdf]

Additional information

Table S1. Values of parameters used in all figures

Figures		β_N	β_Q	γ_N	γ_Q	ρ_0	q	m_S	m_N
Fig. 1 (b)		0.3	0.1	0.1	0.1	0.2	0.2	ND	ND
Fig. 2	(a)	0.8	0.1	0.2	0.2	0.2	---	ND	ND
	(b)	0.4	0.1	0.2	0.2	0.2	---	ND	ND
	(c)	0.1	0.1	0.2	0.2	0.2	---	ND	ND
Fig. 3	(a)	0.3	0.1	0.1	0.1	0.2	0.2	---	---
	(b)	0.3	0.1	0.1	0.1	0.2	0.2	---	---
Fig. 4	(a)	0.3	0.1	0.1	0.1	0.2	0.2	2.0	---
	(b)	0.1	0.3	0.1	0.1	0.2	0.2	2.0	---
	(c)	0.3	0.1	0.1	0.1	0.2	0.8	2.0	---
Fig. 5	(a)	0.3	0.1	0.1	0.1	0.2	---	10.0	10.0
	(b)	0.3	0.1	0.1	0.1	0.2	---	10.0	10.0
	(c)	---	0.1	0.1	0.1	0.2	0.2	10.0	10.0
	(d)	---	0.1	0.1	0.1	0.2	0.2	10.0	10.0
Fig. 6	(a)	0.3	0.1	0.1	0.1	0.2	0.4	0	0
	(b)	0.3	0.1	0.1	0.1	0.2	0.4	10.0	0
	(c)	0.3	0.1	0.1	0.1	0.2	0.4	10.0	10.0
	(d)	0.3	0.1	0.1	0.1	0.2	0.4	ND	ND

ND: No definition.

---: Values are changed (axis)

Figure S1

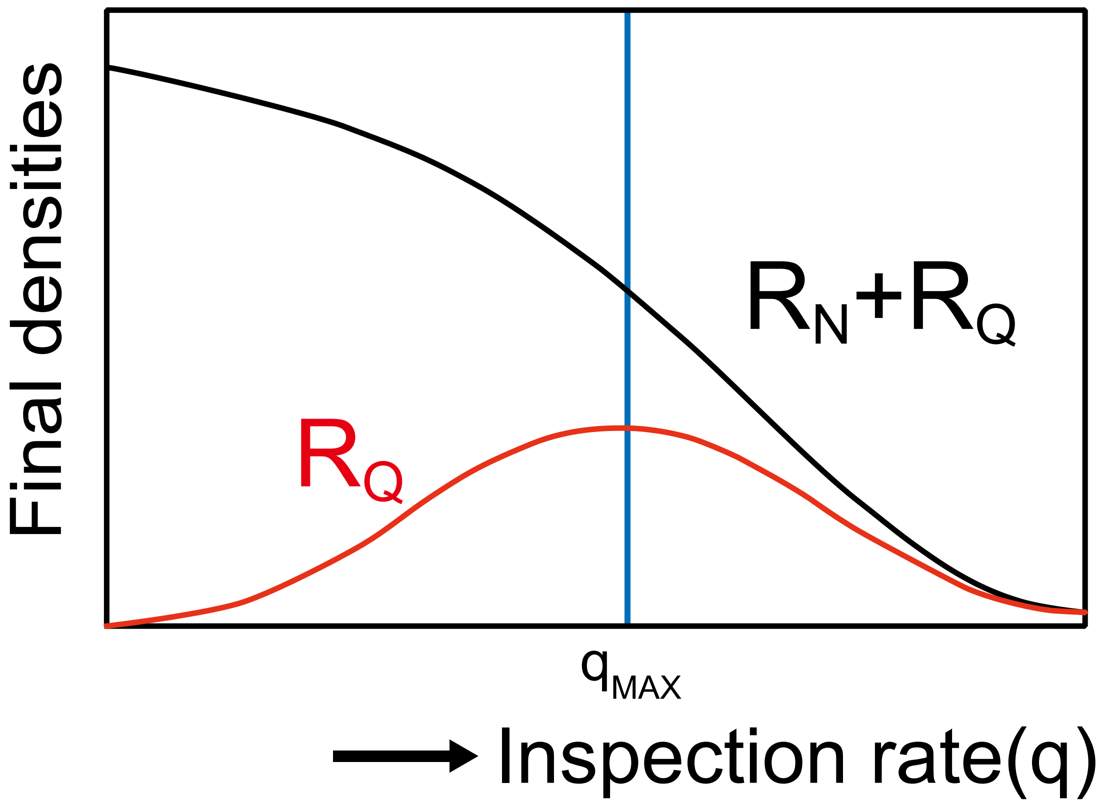

Fig. S1. Schematical illustration of the result obtained by both mean-field theory and random-walk simulation. The population size (density) of quarantined agent (Q) has the maximum at $q = q_{\mathrm{MAX}}$. For $q > q_{\mathrm{MAX}}$, the density of agent Q decreases in spite of increasing the number of tests.
